# La perforation stercorale du côlon: à propos d'un cas et revue de la littérature

**DOI:** 10.11604/pamj.2015.22.249.8114

**Published:** 2015-11-17

**Authors:** Ammar Mahmoudi, Mezri Maâtouk, Faouzi Noomen, Mohamed Nasr, Khadija Zouari, Abdelaziz Hamdi

**Affiliations:** 1 Service de Chirurgie Générale et Digestive, CHU Fattouma Bourguiba de Monastir, Tunisie; 2 Service d'imagerie Médicale, CHU Fattouma Bourguiba de Monastir, Tunisie

**Keywords:** Perforation stercorale, côlon, péritonite, résection, colostomie, intervention de Hartmann, perforation stercoral, colon, peritonitis, resection, colostomy, Hartmann procedure

## Abstract

Affection rare, la perforation stercorale du côlon touche des malades âgés souvent fragiles ayant une longue histoire de constipation chronique et sévère. Elle constitue une urgence chirurgicale dont le pronostic, souvent sombre, dépend du terrain et de la rapidité de la prise en charge. Nous rapportons le cas d'une perforation stercorale du côlon survenu chez une patiente âgée de 74 ans. La symptomatologie clinique était celle d'une péritonite aiguë évoluant depuis quatre jours. Le diagnostic n’était posé qu'en peropératoire. Le geste avait consisté en une intervention de Hartmann. Les suites étaient malheureusement marquées par un état de choc septique résistant aboutissant au décès de la patiente à J 2 postopératoire. Le diagnostic de perforation stercorale du côlon, souvent difficile et retardé, doit être connu par tous les médecins qui prennent en charge une population de patients de plus en plus âgés.

## Introduction

Affection rare, la perforation stercorale du côlon touche des malades âgés souvent fragiles ayant une longue histoire de constipation chronique et sévère. Elle constitue une urgence chirurgicale dont le pronostic, souvent sombre, dépend du terrain et de la rapidité de la prise en charge. Nous rapportons le cas d'une perforation stercorale du côlon survenu chez une patiente âgée de 74 ans dont le diagnostic n’était posé qu'en peropératoire et nous essayons de rappeler la physiopathologie, les caractéristiques cliniques ainsi que les modalités thérapeutiques de cette entité.

## Patient et observation

Patiente âgée de 74 ans, diabétique, hypertendue sous traitement médical, avec une longue histoire de constipation chronique qui avait consulté pour des douleurs abdominales depuis 4 jours. Il n′y avait pas de nausée, pas de vomissement et pas d′arrêt des matières et des gaz. La température était à 38,5 °C. Sur le plan hémodynamique, il y avait une tendance au collapsus. La palpation abdominale avait trouvé une défense abdominale généralisée à maximum au niveau de la fosse iliaque gauche et hypogastrique. Le toucher rectal avait provoqué des douleurs dans le cul-de-sac de Douglas sans masse palpable. Sur le plan biologique, il existait un syndrome infectieux et inflammatoire avec 22 000 leucocytes/mL et une CRP à 345 mg/L. La radiographie de l′abdomen sans préparation n'avait pas montré de niveau hydroaérique ou de pneumopéritoine. Le scanner abdominopelvien avait objectivé un épanchement péritonéal modéré, associé à une importante infiltration péritonéale sans pneumopéritoine ni abcès profond. La patiente a été mise sous antibiothérapie (Claforan+ Flagyl+ Genta) et opérée par une laparotomie médiane après une brève réanimation. L′exploration de la cavité péritonéale avait mis en évidence une péritonite généralisée avancée avec présence de fausses membranes et d′un liquide purulent, prélevé pour examen bactériologique. Il a été constaté une perforation de la face antérieure de la charnière recto-sigmoïdienne, sur le bord antimésentérique, avec un fécalome très dur impacté dans celle-ci. Après une toilette péritonéale abondante, il lui a été réalisé une intervention de Hartmann avec une résection du colon perforé, une fermeture du moignon rectal, et abouchement du colon proximal en stomie. L'intervention s'est terminée par un drainage de la cavité péritonéale. La pièce opératoire était envoyée pour examen anatomopathologique. Les suites étaient marquées par un état de choc septique résistant aboutissant au décès de la patiente à J 2 postopératoire. L′examen anatomopathologique de la pièce opératoire n'avait pas montré de tumeur, de pathologie inflammatoire colique, de diverticulose, ni de lésion ischémique.

## Discussion

Décrite pour la première fois par Berry en 1894 [1], la perforation stercorale du côlon, ou sur fécalome, est une complication très rare de la constipation qui constitue une affection très fréquente, bénigne et relevant le plus souvent d′un traitement médical. Une perforation du côlon est rare, et est habituellement causée par une diverticulite, un traumatisme, une tumeur maligne, une colite amibienne, une colite ischémique, ou une rectocolite ulcéro-hémorragique. La perforation stercorale du côlon représente 3,2% de l′ensemble des perforations coliques [2]. À ce jour, à notre connaissance, moins de 180 cas de perforation stercorale du côlon ont été rapportés dans la littérature.

La perforation stercorale du côlon touche essentiellement les sujets âgés souvent fragiles. C’était le cas de notre patiente qui avait 74 ans et ayant des comorbidités. Elle peut aussi survenir exceptionnellement chez des malades jeunes [3, 4]. Le point commun de tous les cas décrits dans la littérature est une constipation chronique, très ancienne, sévère et répondant mal aux traitements médicaux [5–7]. Notre patiente avait une longue histoire de constipation opiniâtre. Dans plus de 90% des cas, les perforations sont situées sur le sigmoïde ou le haut rectum. Dans notre cas aussi, la perforation siégeait sur la face antérieure de la charnière recto-sigmoïdienne. Différentes hypothèses semblent pouvoir expliquer la fragilité de la jonction recto-sigmoïdienne à savoir l'angulation de la charnière recto-sigmoïdienne réalisant un aspect en « pseudo-sphincter », une vascularisation précaire du bord antimésentérique et une sensibilité élevée aux contraintes mécaniques. Ceci explique que les perforations se situent toujours sur le bord antimésentérique du côlon. La présence d'un ralentissement du transit iatrogène ou idiopathique, le siège le plus fréquent des fécalomes sur le côlon distal et la pression intra-luminale qui y est plus importante, augmentent la pression sur les capillaires sous-muqueux et ainsi les phénomènes ischémiques pariétaux [6].

Quoique la physiopathologie exacte de la perforation stercorale n'est pas encore bien élucidée, il semblerait que les phénomènes mécaniques au contact de fécalomes pétrifiés vont créer une ulcération muqueuse qui va induire une souffrance pariétale colique par ischémie, résultant de la compression entre le fécalome et le pelvis lors de longs efforts de défécation [5, 6]. La rupture de la continuité de la paroi colique peut être facilitée par une faiblesse de la musculeuse qui peut lui être associée [6]. Les tableaux cliniques des perforations stercorales du côlon sont très variables et polymorphes. La forme typique est celle d'une perforation d′un organe creux réalisant un tableau de péritonite aiguë avec une défense voire une contracture abdominale, un syndrome inflammatoire biologique et un pneumopéritoine radiologique [4, 6, 8, 9]. La fréquence des formes peu symptomatiques et trompeuses, car la longue histoire de constipation chronique peut faire négliger les plaintes de douleur abdominale, et l'absence de pneumopéritoine radiologique rendent le diagnostic positif souvent très difficile [1, 2, 5, 10]. Malgré que la clinique était celle d'une péritonite aiguë, chez notre patiente, le diagnostic de la perforation stercorale du côlon n'a pas été évoqué en préopératoire vu l'absence de pneumopéritoine radiologique et la rareté de cette entité. L′examen le plus utile pour le diagnostic de perforation stercorale du côlon est le scanner abdomino-pelvien qui peut montrer une discontinuité de la paroi intestinale en regard d'une distension fécale de la lumière colique [11]. Le diagnostic différentiel de la perforation stercorale du côlon se pose avec les autres causes de perforation colique à savoir les lésions néoplasiques, diverticulaires, inflammatoires, infectieuses, ischémiques ou traumatiques [5, 12]. La perforation stercorale du côlon constitue une urgence chirurgicale. Une fois le diagnostic posé, une réanimation et une antibiothérapie doivent être instaurées en urgence. L'antibiothérapie doit être adaptée au résultat des prélèvements bactériologiques peropératoires. Le geste le plus recommandé est une intervention de Hartmann avec drainage de la cavité abdominale [4, 6–8, 13]. Une extériorisation sur baguette du côlon perforé en stomie est possible [2, 8], mais une biopsie doit être systématique afin d′écarter les autres diagnostics, principalement celui de lésion néoplasique. Une résection colique emportant le colon perforé associée à une anastomose colorectale peut être effectuée d′emblée sous couvert ou non d'une stomie en cas de faible contamination péritonéale [5]. Selon l′importance des lésions et la distension du côlon d′amont, la résection peut être plus étendue allant jusqu′à la colectomie subtotale avec une anastomose iléo-rectale. Dans tous les cas, une évacuation du fécalome doit être réalisée en même temps opératoire ainsi qu'une toilette péritonéale abondante. Une coloscopie peropératoire est préconisée par certains auteurs à la recherche d′ulcérations stercorales afin que la résection colique emporte celles-ci [5]. Dans notre cas, le geste réalisé était une intervention de Hartmann associée à un drainage de la cavité péritonéale. Le pronostic de la perforation stercorale du côlon est très sombre avec un taux de mortalité élevé et une morbidité importante. Ceci est du au retard diagnostic et par conséquent de prise en charge du fait de la difficulté diagnostique, et au terrain sur lequel elle survient avec des patients âgés ayant des comorbidités et en très mauvais état général.

La mortalité secondaire à une perforation stercorale est très élevée et est variable selon les séries atteignant 35% dans la littérature [7, 14] et allant jusqu′à plus de 50% pour certains auteurs [9]. Dans notre cas, l’évolution était fatale malgré une prise en charge adaptée. Cette évolution est due aux antécédents de la patiente et au retard de la prise en charge puisque la symptomatologie évoluait depuis quatre jours. Chez les malades survivants, il faut s′efforcer de lutter contre la constipation par un régime alimentaire et des traitements médicamenteux. Après une intervention de Hartmann, la fermeture de la colostomie doit être prudente et réalisée dans des délais longs, allant jusqu′à 18 mois pour certains auteurs [5].

## Conclusion

La perforation stercorale du côlon est une affection rare qui touche des malades souvent fragiles ayant une longue histoire de constipation chronique et sévère. Le diagnostic, souvent difficile et retardé, doit être connu par tous les médecins qui prennent en charge une population de patients de plus en plus âgés. La perforation stercorale du côlon constitue une urgence chirurgicale dont le pronostic, souvent sombre, dépend du terrain et de la rapidité de la prise en charge.
